# Sympathetic activation of white adipose tissue recruits neutrophils to limit energy expenditure

**DOI:** 10.21203/rs.3.rs-6414640/v1

**Published:** 2025-04-16

**Authors:** Seunghwan Son, Cindy Xu, Janice Jang, Maddox Dinh, Yuliya Skorobogatko, Haipeng Fu, Joseph M. Valentine, Garam An, Wei Ying, Ruth T. Yu, Michael Downes, Ronald M. Evans, Alan R. Saltiel

**Affiliations:** 1 Division of Endocrinology and Metabolism, Department of Medicine and Pharmacology, University of California San Diego, San Diego, CA, USA; 2 Gene Expression Laboratory, Salk Institute for Biological Studies, San Diego, CA, USA

## Abstract

Adipose tissue maintains energy homeostasis by storing lipids during nutrient surplus and releasing them through lipolysis in times of energy demand. While lipolysis is essential for short term metabolic adaptation, prolonged metabolic stress requires adaptive changes that preserve energy reserves. Here, we report that β-adrenergic activation of adipocytes induces a transient and depot-specific infiltration of neutrophils into white adipose tissue (WAT), particularly in lipid-rich visceral WAT. Neutrophil recruitment requires the stimulation of both lipolysis and p38 MAPK activation in adipocytes. Recruited neutrophils locally secrete IL-1β, which suppresses lipolysis and limits excessive energy expenditure. Neutrophil depletion or blockade of IL-1β production increased lipolysis, leading to reduced WAT mass upon repeated β3-adrenergic stimulation. Together, these findings reveal an unexpected role of neutrophil-derived IL-1β in preserving lipid stores during metabolic stress, highlighting a physiological function of innate immune cells in maintaining energy homeostasis.

## Introduction

Adipocytes play a key role in maintaining energy homeostasis in the face of metabolic stress. These cells sense energy status and respond by storing excess nutrients as triacylglycerols during overnutrition, releasing free fatty acids (FFAs) through lipolysis when energy is needed and generating chemokines, cytokines, adipokines and lipokines to regulate other tissues. While lipolysis is essential for metabolic adaptation, unregulated lipolysis can lead to lipotoxicity, insulin resistance, glucose intolerance, chronic inflammation and energy imbalance. Therefore, the tight regulation of lipolysis by hormones and intracellular sensors is a key to metabolic health.

In addition to their role in managing energy metabolism during fasting and feeding cycles, adipocytes respond to metabolic stress by initiating immune responses that modulate insulin and catecholamine sensitivity, tissue remodeling and metabolic adaptation^1–3^. While adipose tissue is normally comprised of resident macrophages^4,5^, obesity produces chronic inflammation that involves the sustained infiltration of pro-inflammatory macrophages and other immune cells^2,6,7^, and transient inflammatory responses have been linked to healthy adipose expansion and browning^8–10^.

Neutrophils are best known for their role as first-line defenders against infection, but remain largely overlooked for their potential involvement in metabolic regulation^11–14^. Circulating neutrophil levels fluctuate with circadian rhythms and metabolic states such as fasting or cold exposure^15–19^, suggesting a close crosstalk between innate immunity and energy balance. Moreover, neutrophils are one of the major sources of IL-1β^20^, a potent pro-inflammatory cytokine that is produced by inflammasome activation. Although IL-1β has been studied for its pathogenic roles in obesity and type 2 diabetes^21,22^, its acute localized actions may also contribute to maintaining tissue homeostasis^23–25^.

While some studies have reported that β-adrenergic stimulation of adipocytes induces a transient inflammatory response^26–28^, the nature and impact of this inflammation, as well as how it is triggered remain unclear. In this study, we identify a previously unrecognized physiological role of neutrophils in regulating energy balance upon β-adrenergic activation of adipocytes. We show that acute injection of a β3-adrenergic agonist or cold exposure induces the transient and depot-specific infiltration of neutrophils into white adipose tissue (WAT), particularly in lipid-rich epididymal WAT. This dramatic neutrophil infiltration is accompanied by a marked drop in resident macrophages. Once recruited, neutrophils secrete the cytokine IL-1β via inflammasome activity. This neutrophil-derived IL-1β limits excessive lipolysis, thereby contributing to the preservation of energy storage during persistent sympathetic activation. Interestingly, neutrophil recruitment requires both adipocyte lipolysis and downstream p38 signaling, while classical chemokine pathways are not sufficient. Together, our findings highlight a previously unappreciated role of immune cells in the homeostatic regulation of adipose tissue metabolism in which neutrophil-driven signals function as a brake on excessive lipid mobilization during sympathetic activation, preserving energy storage.

## Results

### β3 adrenergic activation of adipocytes induces neutrophil infiltration into white adipose tissue

**Extended Fig 1.**
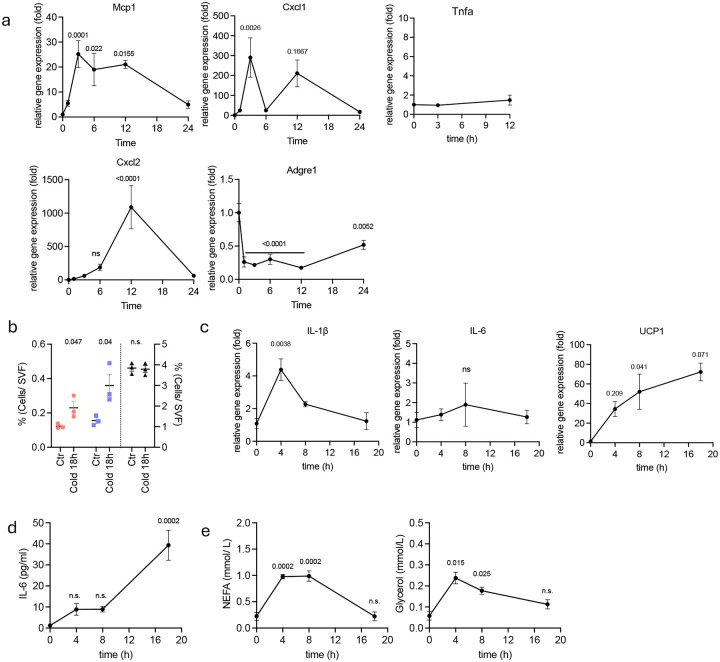
CL-316,243 injection or cold exposure induce neutrophil infiltration into visceral fat along with increased inflammatory cytokine expression. **a**. Inflammatory gene expression in eWAT at indicated time after CL-316,243 injection (n=4–8 per time point). P value for difference compared to time 0h. **b.** Cell frequency of neutrophils, monocytes and macrophages in SVF from iWAT of WT mice exposed to cold for 18 hours. (n=3 per time point). P value for difference compared to time 0h **c**. Inflammatory gene expression in iWAT from mice cold exposed for 18 hours. (n=3–4 per time point). P value for difference compared to time 0h. **d,e**. IL-6, NEFA and glycerol levels from serum at indicated time after cold exposure (n=4 per time point). P value for difference compared to time 0h. a,c,d,e. one-way analysis of variance (ANOVA) with Bonferroni’s post-test, b. two-tailed Student’s *t*-test.

**Fig 1.**
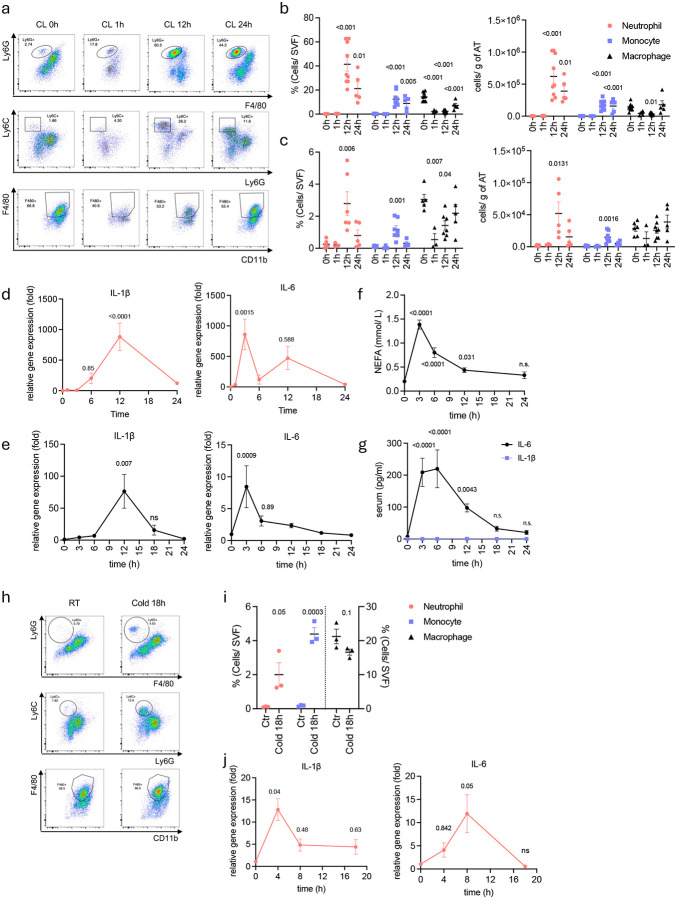
CL-316,243 injection or cold exposure induce neutrophil infiltration into visceral fat along with increased inflammatory cytokine expression. **a**. Representative Fluorescence-Activated Cell Sorting (FACS) data (top: neutrophils, middle: monocytes, bottom: macrophages, gated on CD45+CD11b+Thy1.2-CD19-) and **b,c.** cell frequency/ absolute numbers of neutrophils, monocytes and macrophages in SVF from eWAT (b) or iWAT (c) of WT mice injected with CL-316,243 (0.5 mg/kg) for indicated time (CL 0h: n=10, CL 1h: n=4, CL 12h: n=9, CL 24h: n=5). P value for difference compared to time 0h. **d,e**. Inflammatory gene expression in eWAT (d) and iWAT (e) at indicated times after CL-316,243 injection (n=4–8 per time point). P value for difference compared to time 0h. **f,g**. NEFA, IL-6 and IL-1β levels from serum at indicated times after CL-316,243 injection (n=8 per time point). P value for difference compared to time 0h. **h.** Representative Fluorescence-Activated Cell Sorting (FACS) data (top: neutrophils, middle: monocytes, bottom: macrophages, gated on CD45+CD11b+Thy1.2-CD19-) and **i.** cell frequency/ absolute numbers of neutrophils, monocytes and macrophages in SVF from eWAT of WT mice exposed to cold for 18 hours. (n=3 per time point). P value for difference compared to time 0h. **j.** Inflammatory genes expression in eWAT after 18 hours of cold exposure. (n=3–4 per time point). P value for difference compared to time 0h. b-g. one-way analysis of variance (ANOVA) with Bonferroni’s post-test, I, j. one-way analysis of variance (ANOVA) with Turkey’s post-test.

Previous studies have hinted that both cold exposure and β-adrenergic activation of adipocytes induce changes in the composition of immune cells in adipose tissue^26–29^. However, the specific nature of this change, how it is triggered, and its overall impact on metabolic health remain unclear. We characterized innate immune cell dynamics in white adipose tissue of mice after administration of the β3-selective adrenergic agonist CL-316,243 to specifically mimic sympathetic activation of adipocytes. We observed a substantial influx of neutrophils into epididymal WAT (eWAT), which peaked at 12 hours after injection (Fig 1a, b). Monocyte infiltration was also observed, but at levels significantly lower than seen with neutrophils. Interestingly, there was no increase in macrophage infiltration, although resident macrophage number substantially declined within 1 hour of CL-316,243 injection and gradually recovered by 24 hours (Fig 1a, b). Similar changes in immune dynamics occurred in inguinal WAT (iWAT), although neutrophil infiltration levels were lower than those observed in eWAT (Fig 1c). We evaluated inflammatory gene expression in both white adipose depots upon CL-316,243 treatment. Intriguingly, IL-1β and CXCL2 mRNA levels peaked at 12 hours, in concert with neutrophil infiltration, while IL-6, CCL2 and CXCL1 gene expression peaked earlier (Fig 1d, Extended Fig 1a), suggesting distinct cellular sources for these inflammatory mediators. Moreover, there was no increase in levels of TNFɑ, which plays a key role in adipose tissue inflammation during obesity^30^ (Extended Fig 1a). Consistent with lower neutrophil infiltration, inflammatory gene expression in iWAT was lower than observed in eWAT (Fig 1e). While IL-6 and NEFAs were elevated in serum after CL injection, IL-1β remained undetectable (Fig 1f, g).

As acute cold exposure produces increased sympathetic activation of adipose tissue via release of epinephrine and norepinephrine^31,32^, we also examined changes in immune cell dynamics after cold exposure of mice. Acute cold exposure induced neutrophil infiltration into both eWAT (Fig 1h, i) and iWAT (Extended Fig 1b). Similar to what was observed with CL-316,243 treatment, mRNAs encoding inflammatory cytokines IL-1β and IL-6 were increased upon cold exposure in eWAT (Fig 1j), while only IL-1β did in iWAT (Extended Fig 1c). In addition, IL-6, NEFA and glycerol levels were elevated in serum after cold exposure, although not to the extent observed after CL-316,243 injection (Extended Fig 1d, e).

**Extended Fig 2.**
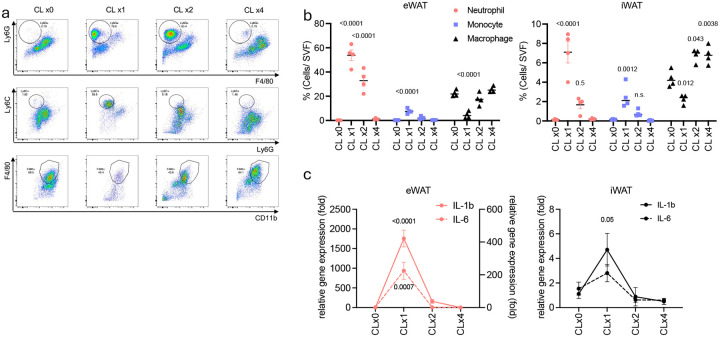
Repeated CL-316,243 injection does not induce sustained neutrophil infiltration. **a**. Representative Fluorescence-Activated Cell Sorting (FACS) data (top: neutrophils, middle: monocytes, bottom: macrophages, gated on CD45+CD11b+Thy1.2-CD19-) and **b.** cell frequency of neutrophils, monocytes and macrophages in SVF from eWAT or iWAT of WT mice injected with CL-316,243 (0.5 mg/kg) for indicated time (CLx1: 1 day, CLx2: 2 days, CLx4: 4 days, n=4 per group). P value for difference compared to time 0h. **c**. Inflammatory gene expression in eWAT or iWAT from mice injected with CL-316,243 for indicated time. (left Y axis: IL-1β, right Y axis: IL-6. n=3–4 per time point). P value for difference compared to time 0h. b,c,e,f,g,h. one-way analysis of variance (ANOVA) with Bonferroni’s post-test.

Like long term cold exposure, repeated CL-316,243 injection is known to induce iWAT browning^33,34^. To assess whether this process is associated with sustained neutrophil infiltration, we administered CL-316,243 for up to four consecutive days. The increase in neutrophil infiltration and elevated inflammatory cytokine gene expression peaked after a single injection, and declined thereafter with only a minimal response by the 4^th^ injection (Extended Fig 2a, b). While this may be a result of desensitization of β3 adrenergic signaling in adipocytes^35^, these data further suggest that visceral adipose tissue is the main target of neutrophil action among white adipose tissues.

### Beta adrenergic-induced neutrophil infiltration is dependent on lipolysis in adipocytes

**Extended Fig 3.**
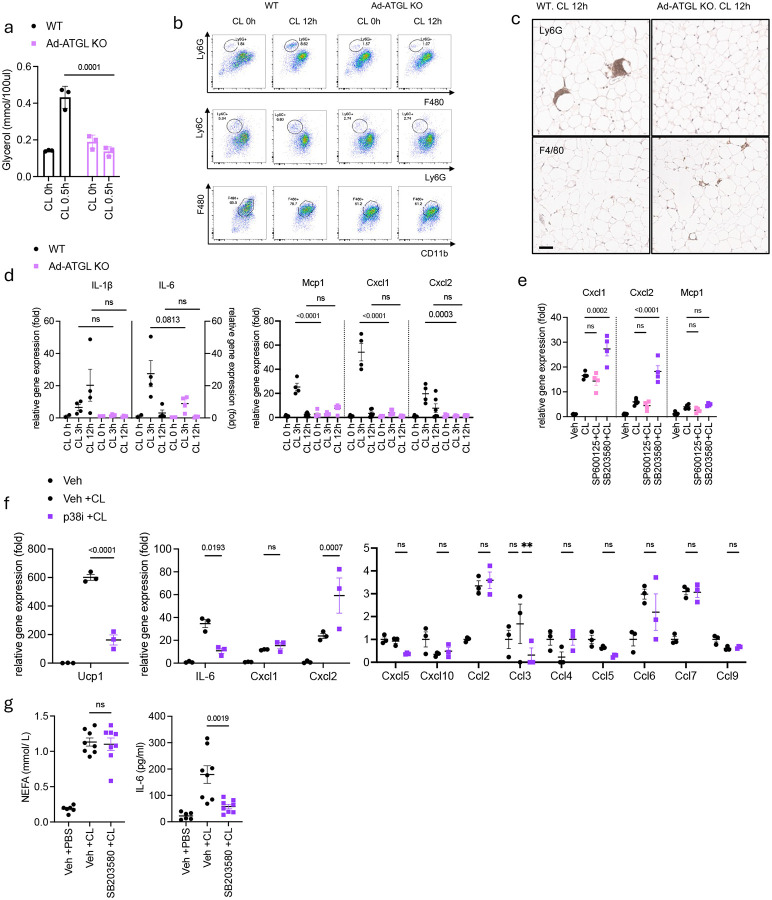
A Lipolysis-p38 axis dependent factor recruits neutrophils into white adipose tissue upon β3 adrenergic activation. **a**. Glycerol levels from serum of WT or ad-ATGL KO mice after CL-316,243 (0.5 mg/kg) injection (n=3 per group). P value for difference between WT and ad-ATGL KO. **b**. Representative Fluorescence-Activated Cell Sorting (FACS) data (top: neutrophils, middle: monocytes, bottom: macrophages, gated on CD45+CD11b+Thy1.2-CD19-) in SVF from iWAT of WT or ad-ATGL KO mice injected with CL-316,243 (0.5 mg/kg) for 12 hours. **c.** Representative Ly6G (top) and F4/80 (bottom) staining image of eWAT sections from WT or ad-ATGL KO mice 12 hours after CL-316,243 injection. scale: 100 μm **d**. Inflammatory gene expression in iWAT from WT or ad-ATL KO mice injected with CL-316,243 for indicated time. (left Y axis: IL-1β, right Y axis: IL-6. n=4 per time point, IL-1β, IL-6 CL 0h: n=2). P value for difference between WT and ad-ATGL KO at each time point. **e,f**. Chemokine expression in WT ppdivs pretreated 30 min with JNK (SP600125, 10 uM) or p38 (SB203580, 10 uM) inhibitor, followed by CL-316,243 (1 uM) for 3 hours. (n=3–4 per group). P value for difference compared to CL-316,243 alone. **g**. NEFA and IL-6 levels from serum 6h after CL-316,243 injection (Veh+PBS: n=6, Veh+CL, SB203580: n=8). P value for difference between CL and inhibitors+CL. a,d. two-way analysis of variance (ANOVA) with Bonferroni’s post-test. e,f,g. one-way analysis of variance (ANOVA) with Bonferroni’s post-test.

**Fig 2.**
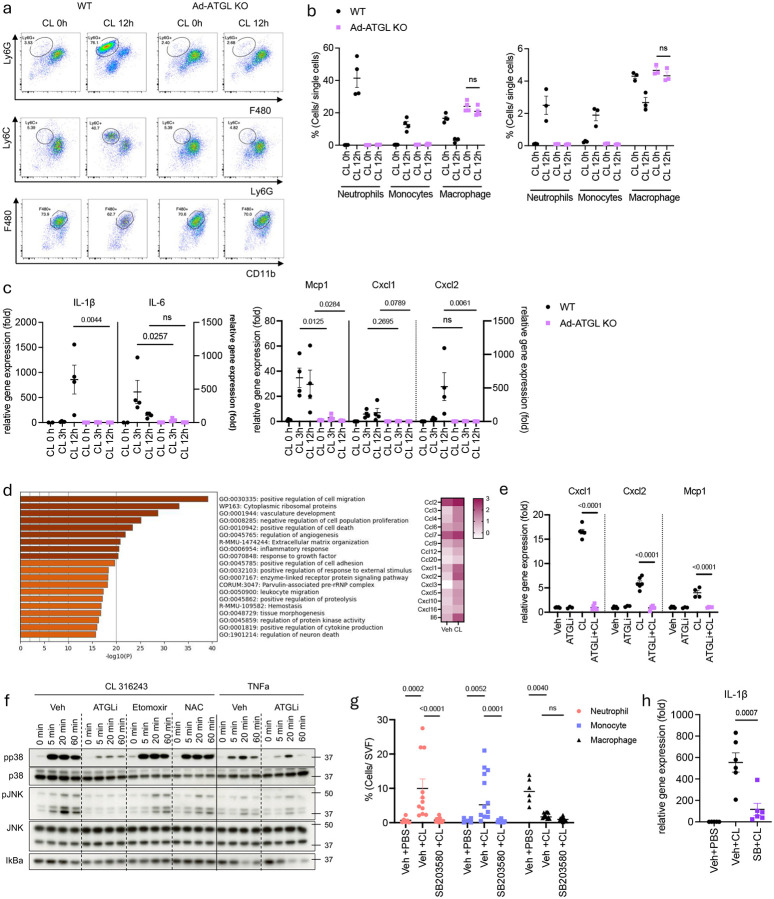
A Lipolysis-p38 axis dependent factor recruits neutrophils into white adipose tissue upon β3 adrenergic activation. **a**. Representative Fluorescence-Activated Cell Sorting (FACS) data (top: neutrophils, middle: monocytes, bottom: macrophages, gated on CD45+CD11b+Thy1.2-CD19-) and **b.** Cell frequency of neutrophils, monocytes and macrophages in SVF from eWAT (left, n=4) or iWAT (right, n=3) of WT or ad-ATGL KO mice injected with CL-316,243 (0.5 mg/kg) for 12 hours. P value for difference between WT and ad-ATGL KO. **c**. Inflammatory gene expression in eWAT from WT or ad-ATL KO mice injected with CL-316, 243 for indicated time. (left Y axis: IL-1β, MCP1, right Y axis: IL-6, Cxcl1, Cxcl2. n=4 per time point, IL-1β, IL-6 CL 0h: n=2). P value for difference between WT and ad-ATGL KO. **d.** Enriched clustered ontology analysis (left) and expression differences of genes involved (right) from WT primary preadipocytes differentiated in vitro (ppdivs) treated with CL-316,243 (1 uM, 3 hours) **e**. Inflammatory gene expression in WT ppdivs pretreated 30 min with Atglistatin (ATGLi, 20 uM), followed by CL-316,243 (1 uM) for 3 hours. (n=4 per group). P value for difference between treatment. **f.** Activation of p38 and JNK phosphorylation by CL-316,243 (1 uM) along with inhibitors (Atglistatin: 20 uM, Etomoxir: 10 uM, NAC: 10 mM. 30 min pretreatment) in 3T3L1 adipocytes. **g.** Cell frequency of neutrophils, monocytes and macrophages in SVF from eWAT of WT mice injected with SB203580 (25 mg/kg), followed by CL-316,243 (0.5 mg/kg) for 6h. (Veh+PBS: n=6, Veh+CL: n=11, SB203580+CL: n=12). P value for difference between CL and inhibitors+CL. **h**. IL-1β gene expression in eWAT from WT mice injected with SB203580 (25 mg/kg), followed by CL-316,243 (0.5 mg/kg) for 6h. (veh+PBS: n=5, veh+CL, SB+CL: n=6 per group). P value for difference between CL and inhibitors+CL. b,c,g. two-way analysis of variance (ANOVA) with Bonferroni’s post-test. e,h. one-way analysis of variance (ANOVA) with Bonferroni’s post-test.

Because the β-3 adrenergic receptor targeted by CL-316,243 is predominantly expressed in adipocytes and no other cells found in adipose tissue^36–38^, we postulated that neutrophil infiltration is the result of a chemotactic factor(s) originating from adipocytes upon their activation. The β-3 adrenergic receptor signals via the cAMP/PKA pathway to increase lipolysis via phosphorylation of hormone sensitive lipase (HSL), as well as phosphorylation of transcription factors to increase expression of a number of genes, including several encoding chemokines, cytokines and adipokines^26,39,40^. To characterize the process by which adipocytes recruit neutrophils into WAT during cold exposure or CL-316,243 treatment, we first assessed the role of lipolysis. We created Adipoq-cre-driven adipocyte-specific ATGL-KO mice (ad-ATGL KO); these mice were unable to mount a lipolytic response after CL-316,243 injection (Extended Fig 3a). Surprisingly, ATGL KO showed no neutrophil infiltration into eWAT or iWAT after CL-316,243 injection (Fig 2a, b, Extended Fig 3b, c). In addition, macrophage number did not decline in KO mice after CL-316,243 injection. Our previous studies revealed that β-adrenergic activation can induce the expression and release of several chemokines from isolated adipocytes that might influence neutrophil migration^35^, leading us to assess whether these changes were dependent on lipolysis *in vivo* (Fig 2c, Extended Fig 3d). We demonstrated that the chemokines CXCL1, CXCL2 and CCL2 were induced within 3 hours of CL-316,243 injection, well before neutrophil infiltration, and thus likely originated from adipocytes. Interestingly, the induction of these cytokines by CL-316,243 was markedly reduced in ad-ATGL KO mice (Fig 2c).

To confirm that the chemokines induced upon β3-adrenergic activation in mice were from adipocytes, we conducted RNA-seq analysis on primary adipocytes differentiated from iWAT preadipocytes *in vitro* before and after CL-316,243 treatment. The expression of several genes encoding cell migration and inflammatory response proteins was observed in response to β-adrenergic activation (Fig 2d). Because neutrophil migration depended on adipocyte lipolysis, we investigated whether increased expression of these chemokines could be blocked by lipolysis inhibition *in vitro* (Fig 2c). Treatment of primary adipocytes with the selective ATGL inhibitor Atglistatin abolished the induction of CXCL1, CXCL2, and CCL2 expression by CL-316,243 (Fig 2e). Moreover, blocking lipolysis suppressed phosphorylation of p38 and JNK induced by CL-316,243 in 3T3-L1 adipocytes, independently of ROS or fatty acid oxidation, suggesting these pathways might be required for regulated chemokine expression (Fig 2f). However, inhibiting the p38 or JNK pathway with specific inhibitors failed to reduce the induction of CXCL1, CXCL2 and CCL2 mRNA expression by CL-316,243 in primary adipocytes (Extended Fig 3e, f), although p38 inhibition suppressed the induction of UCP1 and IL-6 expression (Extended Fig 3f), as previously shown^41,42^.

To explore further the importance of adipocyte MAP kinase pathways in neutrophil infiltration after β-adrenergic activation of adipocytes, we administered the p38 inhibitor SB203580 *in vivo*. p38 inhibition effectively blocked CL-316,243-induced neutrophil infiltration and IL-1β production in eWAT (Fig 2g, h). SB203580 had no effect on lipolysis, but blocked the increase in circulating IL-6 levels produced by CL-316,243 (Extended Fig 3g), aligning with our previous reports of IL-6 regulation via the p38 pathway^42^. Collectively, these data show that CL-316,243-induced neutrophil infiltration into WAT depends on both lipolysis and subsequent p38 activation. These findings indicate that the canonical chemokines CXCL1, CXCL2 and CCL2 do not appear to be sufficient for neutrophil infiltration, despite being known chemoattractants for these immune cells^43^, suggesting the role of an additional adipocyte chemotactic factor that must require both lipolysis and activation of the p38 pathway for increased expression and release.

### Neutrophils are the source of IL1 production in adipose tissue upon β-adrenergic activation of adipocytes

**Extended Fig 4.**
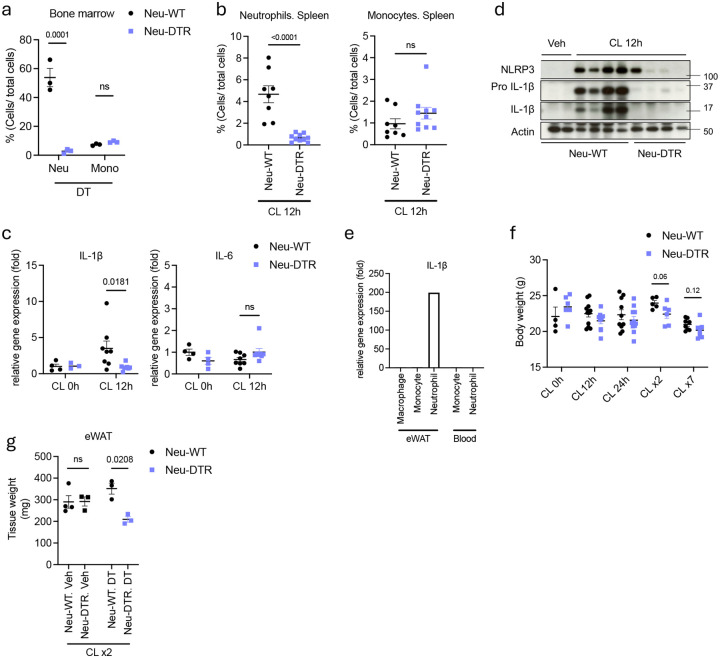
Infiltrated neutrophils are the source of IL-1β in adipose tissue upon β3 adrenergic activation and suppress excessive energy expenditure. **a.** Cell frequency of neutrophils and monocytes in bone marrow from mice injected with diphtheria toxin (DT, 20 pg/kg) only for 24 hours (n=3 per group). P value for difference between group. **b.** Cell frequency of neutrophils and monocytes in spleen from mice injected with CL-316,243 after neutrophil depletion using DT (Neu-WT: n=8, Neu-DTR: n=10). P value for difference between. **c.** Inflammatory genes expression in iWAT from Neu-WT or Neu-DTR mice injected with CL-316,243 for 12 hours (CL 0h: n=3–4, CL 12h: n=7–8). P value for difference between Neu-WT and Neu-DTR. **d**. Pro and active form of IL-1β in whole eWAT lysate of Neu-WT and Neu-DTR mice injected with CL-316,243 for 12 hours. **e.** IL-1β gene expression level of macrophage, monocyte and neutrophil in SVF cells from WT mice eWAT and blood injected with CL-316,243 for 12 hours. Each population is separated through cell sorting (n=3 pooled together for cell sorting). **f.** Body weight of Neu-WT and Neu-DTR mice injected with CL-316,243 for indicated time. **g.** Tissue weight of eWAT from Neu-WT and Neu-DTR mice injected with or without DT along with CL-316,243 for 2 days (n=3 per group). P value for difference between Neu-WT and Neu-DTR. a. two-way analysis of variance (ANOVA) with Bonferroni’s post-test. b,f. student’s *t*-test. c,g one-way analysis of variance (ANOVA) with Bonferroni’s post-test.

**Fig 3.**
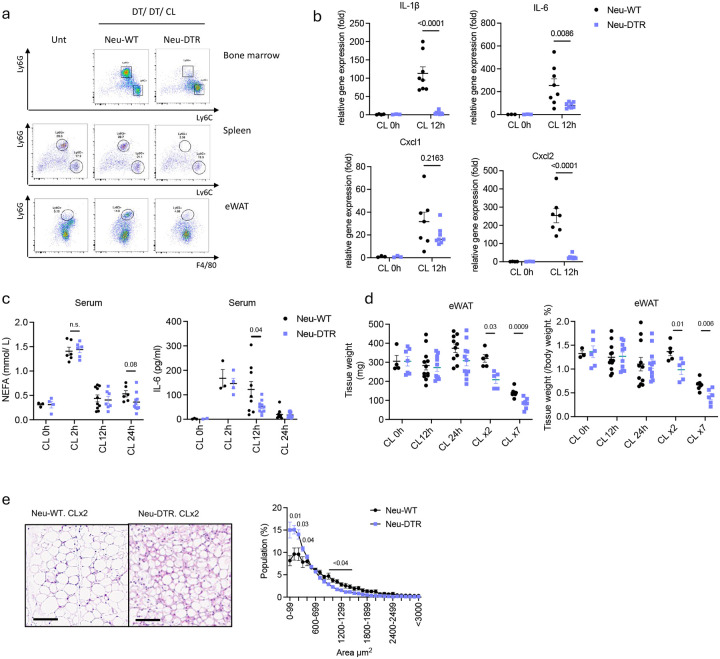
Infiltrated neutrophils are the source of IL-1β in adipose tissue upon β3 adrenergic activation and suppress excessive energy expenditure. **a.** Representative Fluorescence-Activated Cell Sorting (FACS) data of neutrophils and monocytes from each tissue (top: bone marrow, middle: spleen, bottom: eWAT, gated on CD45+CD11b+Thy1.2-CD19-) of Neu-WT or Neu-DTR mice injected with DT (20 pg/kg), followed by CL-316,243 (0.5 mg/kg) for 12 hours. **b.** Inflammatory gene expression in eWAT from Neu-WT or Neu-DTR mice injected with CL-316,243 for 12 hours (CL 0h: n=3–4, CL 12h: n=7–9). P value for difference between Neu-WT and Neu-DTR. **c.** NEFA and IL-6 levels in serum from Neu-WT or Neu-DTR mice injected with CL-316,243 for indicated time (CLx2: 2 days, CLx7: 7 days injection). (n=4–10 per time point). P value for difference between Neu-WT and Neu-DTR. **d**. Tissue weight of eWAT from Neu-WT or Neu-DTR after injected with CL-316,243 for indicated time (CLx2: 2 days injection, CLx7: 7 days injection, n=4–12). P value for difference between Neu-WT and Neu-DTR. **e.** Representative H&E staining image (left) of eWAT sections from Neu-WT or Neu-DTR mice 5h after second CL-316,243 injection. scale: 100 μm. Quantification of adipocytes size (right, n=5 per group). P value for difference between Neu-WT and Neu-DTR. b. two-way analysis of variance (ANOVA) with Bonferroni’s post-test. c-d. student’s *t*-test.

To investigate the function of neutrophil infiltration of white adipose tissue (WAT) after cold exposure or CL-316,243 treatment, we generated neutrophil-specific diphtheria toxin receptor (Neu-DTR) expressing mice by crossing DTR-floxed mice with MRP8-Cre transgenic mice^44^. Administration of diphtheria toxin (DT) effectively depleted neutrophils in bone marrow and peripheral organs, including the spleen, without affecting the monocyte population both before and after CL-316,243 injection (Extended Fig 4a, b). Neutrophil infiltration was significantly reduced in eWAT of Neu-DTR mice after CL-316,243 injection (Fig 3a). The CL-316,243-dependent increase in inflammatory cytokine and chemokine levels (IL-1β, CXCL2) was blocked in both adipose tissues (Fig 3b, Extended Fig 4c), indicating neutrophils are the source of these cytokines after β3-adrenergic receptor activation. CXCL1 levels were not significantly reduced by neutrophil depletion, indicating that other cells, such as adipocytes, monocytes and macrophages are the source of this chemokine in WAT (Fig 3b). However, there was a complete block of CL-316,243-dependent active IL-1β production in eWAT of Neu-DTR mice (Extended Fig 4d), indicating that neutrophils are the primary source of this cytokine following β-adrenergic activation of adipocytes. The predominant expression of IL-1β in the neutrophil fraction after cell sorting supports this idea (Extended Fig 4e). In addition, IL-1β was significantly elevated in adipose tissue but not detectable in serum, suggesting that it acts as a paracrine factor (Fig 1g). Moreover, both the basal and CL-316,243-dependent increase in lipolysis and circulating IL-6 levels were comparable between wild-type and NeuDTR mice (Fig 3c). Taken together, these data confirm that neutrophils are the main source of adipose tissue IL-1β after cold exposure or CL-316,243 treatment.

As neutrophil infiltration peaks at 12 hours post-injection, we focused on later time points to assess the impact of neutrophil infiltration on WAT upon cold or Cl-316,243 injection. Neu-WT or Neu-DTR mice were injected with CL-316,243 for 2 or 7 days along with DT to maintain neutrophil depletion. Surprisingly, despite comparable fat size between the genotypes at 12 hours post-injection when the initial infiltration of neutrophils peaked, adipose tissue mass was significantly reduced after the second CL-316,243 injection in Neu-DTR compared to WT mice (Fig 3d). Histological analysis revealed smaller adipocytes in neutrophil-depleted mice, indicating enhanced energy expenditure (Fig 3e). Overall body weight remained similar between the groups (Extended Fig 4f). DT alone (Fig 3d; CL 0h) and Neu-DTR mice without neutrophil depletion had WAT sizes comparable to wild-type mice (Extended Fig 4g), confirming that this is a neutrophil-dependent phenotype.

**Extended Fig 5.**
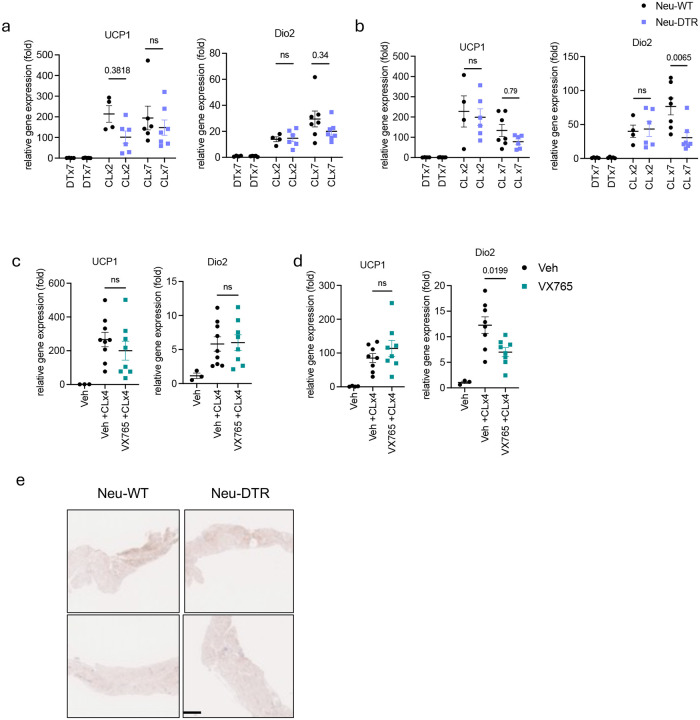
Infiltration of neutrophils into adipose tissue upon β3 adrenergic activation does not affect thermogenic gene expression. **a,b.** Thermogenic gene expression in iWAT (a) and eWAT (b) from Neu-WT or Neu-DTR mice injected with CL-316,243 (0.5 mg/kg) and DT (20 pg/kg) for 2 or 7 days (CL x2: 2 days, CLx7: 7 days, DT: Diphtheria toxin. n=4–7). P value for difference between Neu-WT and Neu-DTR. **c,d.** Thermogenic gene expression in iWAT (c) and eWAT (d) from WT mice injected with vehicle or VX765 (50 mg/kg, twice/ day), along with CL-316,243 for 4 days (Veh: n=3, Veh/ VX765+CL group: n=9,8). P value for difference between vehicle and VX765 group. **e.** Representative UCP1 staining image of iWAT sections from Neu-WT (left) or Neu-DTR (right) mice injected with CL-316,243 for 7 days. a-d. one-way analysis of variance (ANOVA) with Bonferroni’s post-test.

Consecutive daily injection of CL-316,243 induces iWAT browning^33,34^. Although neutrophil infiltration into this depot is much lower than that seen in eWAT, we nevertheless assessed whether neutrophil depletion enhances browning in this paradigm. There were no differences in the induction of thermogenesis-related genes in neutrophil-depleted mice or in mice treated with the capsase 1 inhibitor VX765 to reduce IL-1β production (Extended Fig 5a–e), suggesting neutrophils do not directly modulate iWAT browning. Overall, our findings suggest neutrophils affect visceral but not subcutaneous WAT upon β3-adrenergic activation to conserve energy in adipocytes by driving local IL-1β secretion, limiting further excessive energy expenditure.

### Neutrophil-derived IL-1β suppresses lipolysis to preserve excessive energy usage.

**Extended Fig 6.**
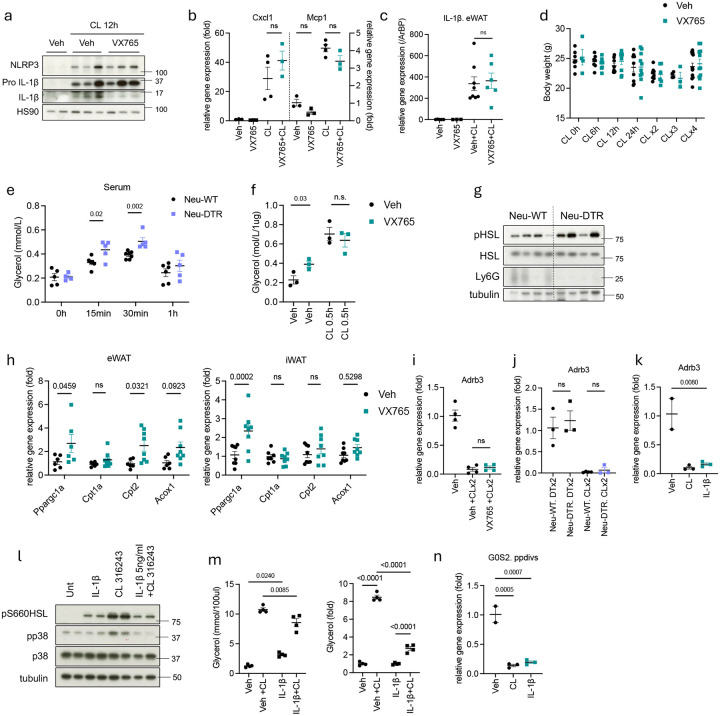
Neutrophil-derived IL-1β suppresses lipolysis by producing catecholamine resistance in adipocytes to preserve excessive energy usage. **a.** Pro and active form of IL-1β in whole eWAT lysate of WT mice injected with vehicle or VX765 (50 mg/kg, twice/day) followed by CL-316,243 injection (0.5 mg/kg) for 12 hours. **b.** Chemokine expression in WT ppdivs pretreated 30 min with VX765, followed by CL-316,243 (1 uM) for 3 hours. (n=3, 4 per group). **c.** IL-1β gene expression in eWAT from WT mice injected with vehicle or VX765, followed by CL-316,243 injection for 12 hours (CL 0h: n=3, CL 12h: n=6,8). P value for difference between vehicle and VX765 group. **d.** Body weight of WT mice injected with either vehicle or VX765 with CL-316,243 for indicated time. **e.** Glycerol levels in serum from Neu-WT or Neu-DTR mice injected with second CL-316,243 for indicated time (n=4–6 per time point). P value for difference between Neu-WT and Neu-DTR. **f.** Glycerol levels in culture media from ex vivo culture of eWAT. eWAT was dissected from WT mice injected with CL-316,243 for 24 hours, along with vehicle or VX765 (n=3 per group). P value for difference between veh and VX765. **g.** Phosphorylation of HSL and PKA substrate in whole eWAT lysate from Neu-WT or Neu-DTR mice 1 hour after injected with second CL-316,243. **h.** Fatty acid oxidation gene expression in both eWAT or iWAT from WT mice 3h post-second CL-316,243 injection, with either vehicle or VX765 (Veh+CLx2: n=6, VX765+CLx2: n=8). P value for difference between vehicle and VX765 group. **i,j.** Beta 3 adrenergic receptor gene expression in eWAT from Neu-DTR mice (j) or WT mice (i) 3h post-second CL-316,243 injection, with either vehicle or VX765 (Ne-DTR: n=4, WT: n=3). P value for difference between different group. **k,n.** Beta 3 adrenergic receptor or G0S2 gene expression in ppdivs treated with CL-316,243 (1 uM, 3h) or IL-1β (5 ng/ml, 3h) (n=2–3 per group). P value for difference compared to vehicle. **l,m.** Primary adipocytes pretreated with IL-1β (5 ng/ml) for 18 hours, followed by CL-316,243 (1 uM) for 0.5 hours. Culture medium was collected to measure released glycerol (m, left). Fold change compared to vehicle or IL-1β (m, right). (n=4 per group). P value for difference between group. Lysate was immunoblotted for phosphorylation of HSL (l). b,c,f,i-k,m,n. one-way analysis of variance (ANOVA) with Bonferroni’s post-test. h. two-way analysis of variance (ANOVA) with Bonferroni’s post-test. d,e. student’s *t*-test.

**Fig 4.**
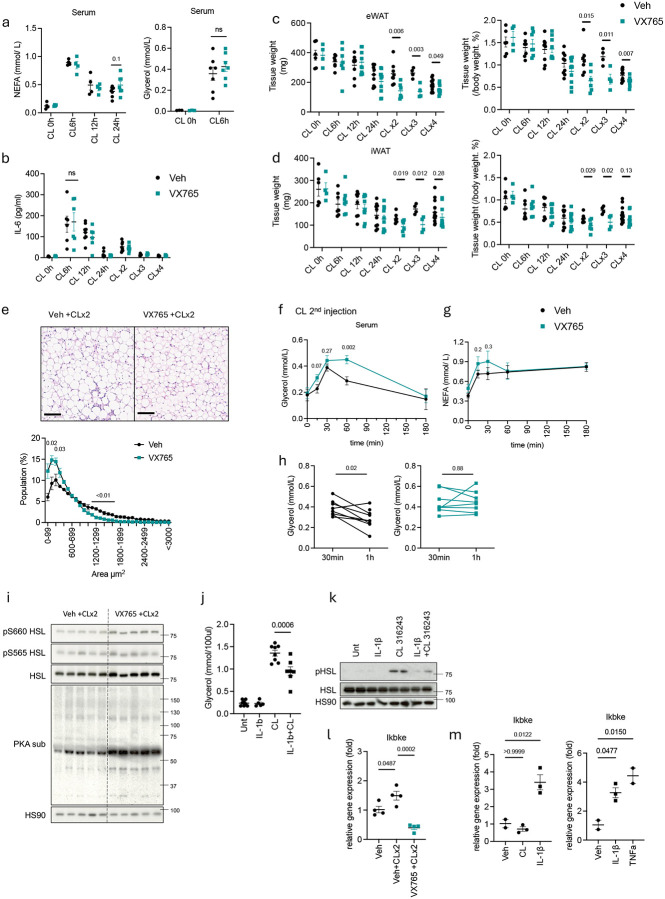
Neutrophil-derived IL-1β suppresses lipolysis by producing catecholamine resistance in adipocytes to preserve excessive energy usage. **a,b.** NEFA, glycerol (a) and IL-6 (b) levels from serum at indicated time after vehicle or VX765 (50 mg/kg, twice/day) along with CL-316,243 injection (0.5 mg/kg, CLx2: 2 days, CLx3: 3 days, CLx4: 4 days injection). (n=4–8 per time point). P value for difference between vehicle and VX765 group. **c,d**. Tissue weights of eWAT (top) or iWAT (bottom) of WT mice injected with either vehicle or VX765 with CL-316,243 for indicated time (n=5–10 per group). P value for difference between vehicle and VX765 group. **e.** Representative H&E staining image of eWAT sections (left) from WT mice 3h after second CL-316,243 injection, with either vehicle or VX765. scale: 100 μm. Quantification of adipocytes size (right, n=5 per group). P value for difference between vehicle and VX765 group. **f,g.** Glycerol and NEFA levels in collected serum from single WT mice at indicated time point, after injecting second CL-316,243 along with vehicle or VX765 (n=8 per group). P value for difference between vehicle and VX765 group. **h.** Individual values of each mouse on serum glycerol and NEFA level at 30 min and 1 hour after second CL-316,243 injection, along with vehicle or VX765. P value for difference between vehicle and VX765 group. **i.** Phosphorylation of HSL and PKA substrate in whole eWAT lysate from WT mice 3 hours after injected with second CL-316,243 along with vehicle or VX765. **j,k.** 3T3L1 adipocytes pretreated with IL-1β (10 ng/ml) for 18 hours, followed by CL-316,243 (1 uM) for 0.5 hours. Culture medium was collected to measure released glycerol (j). Lysate was immunoblotted for phosphorylation of HSL (k). **l.** IKKε gene expression in eWAT from WT mice 1h after second CL-316,243 injection, with either vehicle or VX765 (n=4). P value for difference between vehicle and VX765. **m.** IKKε gene expression in ppdivs (left) and 3T3L1 adipocytes (right) upon IL-1β (5 ng/ml, 3h) or CL-316,243 (1 uM, 3h) or TNFa (5 ng/ml, 3h) treatment (n=2–3). P value for difference between Veh and treatment. a-d,f-h. student’s *t*-test. j-m. one-way analysis of variance (ANOVA) with Bonferroni’s post-test.

As neutrophil depletion completely abolished the appearance of both pro and active IL-β protein in adipose tissue after β-adrenergic activation (Extended Fig 4d), we explored directly whether neutrophil-derived IL-1β is responsible for limiting excessive energy expenditure. We employed the specific caspase 1 inhibitor VX765 to block the processing of active IL-1β^45^. VX765 preadministration effectively blocked CL-316,243-induced IL-1β production by neutrophils in eWAT (Extended Fig 6a) without affecting lipolysis, IL-6 production or chemokine production, ruling out off-target effects (Fig 4a, b, Extended Fig 6b). At 12 hours post-injection, IL-1β mRNA expression and pro–IL-1β protein levels remained comparable between VX765 and vehicle-treated groups, confirming that VX765 did not impact neutrophil infiltration into WAT (Extended Fig 6a, c).

To investigate the effects of neutrophil-derived IL-1β on WAT under cold or CL-316,243 stimulation, we focused on time points beyond 12 hours post-injection when neutrophil infiltration peaks. Similar to what was observed with neutrophil depleted mice, blocking IL-1β production significantly reduced both eWAT and iWAT size following a second CL-316,243 injection (Fig 4c, d), without affecting overall body weight (Extended Fig 6d). Histological analysis of eWAT further confirmed reduced adipocyte size in eWAT of VX765-injected mice (Fig 4e).

Since neutrophil depletion did not alter thermogenic gene expression (Extended Fig 5a–e), we focused on how IL1β might impact WAT lipolysis and fatty acid oxidation. VX765-injected mice showed prolonged glycerol release and slightly increased serum NEFAs upon a second CL-316,243 injection compared to controls (Fig 4f–h). In addition, neutrophil-depleted mice similarly exhibited elevated serum glycerol after the second CL-316,243 injection (Extended Fig 6e). While *ex vivo* cultured eWAT from VX765-treated mice showed higher basal lipolysis, the level of CL-316,243 induced lipolysis was comparable (Extended Fig 6f). eWAT from both VX765-injected or neutrophil-depleted mice showed increased phosphorylation of HSL and other PKA substrates upon a second CL-316,243 injection compared to their respective controls (Fig 4i, Extended Fig 6g). Moreover, blocking IL-1β production with VX765 injection enhanced the expression of fatty acid oxidation genes in both eWAT and iWAT after a second CL-316,243 injection (Extended Fig 6h).

We sought to explore the mechanism by which IL-1β might reduce lipolysis and lipolysis-induced oxidative metabolism. IL-1β treatment inhibited CL-316,243-induced lipolysis and reduced phosphorylation of hormone sensitive lipase (HSL) in both 3T3-L1 adipocytes and primary adipocytes *in vitro* (Fig 4j,k, Extended Fig 6l,m). In primary adipocytes, IL-1β alone produced a small but significant increase in basal lipolysis, which may be due to reduced expression of G0S2, an endogenous inhibitor of ATGL^46^ (Extended Fig. 6n). However, IL-1β pretreatment suppressed CL-316,243–induced lipolysis (Extended Fig. 6m). We previously reported that the protein kinase IKKε reduces cAMP levels by phosphorylating and activating PDE3B, leading to decreased cAMP levels and a subsequent reduction in lipolysis^47^. Notably, a second CL-316,243 injection showed increased IKKε expression in eWAT, which was reduced by IL-1β blockade (Fig 4l). IL-1β treatment upregulated IKKε mRNA expression *in vitro* in both primary and 3T3-L1 adipocytes, whereas CL-316,243 was without effect (Fig 4m). Additionally, while β3-adrenergic receptor (*Adrb3*) expression was unchanged in eWAT of VX765-treated or neutrophil-depleted mice after the second CL-316,243 injection, since β-3 adrenergic agonists alone produce homologous desensitization of the receptor^35^, IL-1β treatment of adipocytes *in vitro* reduced *Adrb3* expression in primary adipocytes (Extended Fig. 6i–k), raising the possibility of β3-adrenergic receptor desensitization as an additional mechanism for IL-1β induced catecholamine resistance. Taken together, these data show that neutrophil-derived IL-1β suppresses lipolysis, leading to prevention of excessive energy usage and promotion of energy storage.

## Discussion

The role of neutrophils in host defense is well established^11,14^; these cells are often the first responders to acute inflammation and can contribute to inflammation resolution by interactions with other immune cells^48,49^. Neutrophils may also play a role in chronic inflammation in various skin diseases, atherosclerosis, diabetes and autoimmune disorders^14,50,51^. We sought to explore their role in acute metabolic stress. Sympathetic activation by cold exposure or injection of a synthetic β-adrenergic agonist resulted in the recruitment of neutrophils to adipose tissue through a process that requires both lipolysis and activation of the p38 MAP kinase pathway in adipocytes. Interestingly, the increase in neutrophils following sympathetic activation of adipocytes was considerably higher in visceral compared to subcutaneous fat, mirroring the differential sensitivity of these depots to inflammation during obesity^52,53^. Once recruited into adipose tissue, neutrophils release the inflammatory cytokine IL-1β via active caspase 1. IL-1β behaves as a paracrine factor to transiently preserve energy balance in adipocytes by limiting lipolysis, thus preventing excessive loss of lipid from visceral fat for sustained survival in the face of catabolic stress.

These findings raise a number of new questions regarding the regulation of energy metabolism in adipocytes. Why is visceral fat specifically targeted by neutrophils? Adipocytes in this depot cells are essentially anabolic with limited capacity for lipid oxidation^54^ and represent a primary site for energy storage and mobilization. In contrast, subcutaneous adipocytes respond to sympathetic activation by undergoing thermogenic remodeling, producing beige adipocytes with increased expression of UCP-1 and other thermogenic genes. Visceral fat lipolysis provides the fuel for thermogenesis in the form of fatty acids^55,56^, suggesting that neutrophil infiltration might be biased towards visceral fat in order to ensure a long-term supply of triglycerides for sustained lipolysis, in the process supporting thermogenesis in beige and brown fat.

A second question concerns the mechanism by which IL-1β represses lipolysis in visceral adipocytes. IL-1β inhibits catecholamine-stimulated lipolysis via reduced phosphorylation of hormone sensitive lipase *in vivo* and *in vitro*. The cytokine activates the NFκB pathway in adipocytes^57–59^, and our previous studies indicate that this pathway can repress catecholamine action through reduced expression of β-3 adrenergic receptor gene expression^35^ as well as activation of the cAMP phosphodiesterase PDE3B via phosphorylation by the NFκB-inducible protein kinase IKKε^47^. Indeed, *in vivo* injection of a β-3 adrenergic agonist indirectly induced the expression of IKKε in visceral adipose tissue; this increase was blocked by caspase 1 inhibition, demonstrating that neutrophil-derived IL-1β induces IKKε in this paradigm. Moreover, treatment of 3T3L1 adipocytes and primary mouse adipocytes with IL-1β produced increased expression of IKKε mRNA and downregulation of the β-3 adrenergic receptor *Adrb3* mRNA. Together these data indicate that IL-1β works through multiple mechanisms to produce catecholamine resistance in adipocytes.

What triggers the recruitment of neutrophils into adipose tissue after sympathetic activation? This phenomenon depends on both increased lipolysis and p38 MAP kinase activation in adipocytes, suggesting that the relevant chemotactic factor requires both pathways for increased synthesis and secretion. Interestingly, classical chemokines such as CXCL1, CXCL2 and CCL2^43^ were not suppressed by p38 inhibition, raising the possibility that these chemokines are not sufficient for neutrophil chemotaxis and that other lipolysis-dependent factors are involved. However, lipolysis alone is unlikely to generate the neutrophil chemoattractant, as p38 inhibition did not affect lipolysis, but blocked neutrophil recruitment. One interesting possibility is that p38 regulates production of lipid mediators whose precursors are produced by lipolysis in adipocytes. One candidate is the arachidonate acid metabolite LTB4 produced by 5-lipoxygenase (5-LOX)^43,60^. LTB4 is a known chemoattractant for neutrophils^61,62^, and 5-LOX inhibition was shown to reduce neutrophil recruitment after CL-316,243, although in this case LTB4 was thought to originate from SVF cells^28^. Moreover, increased p38 activity can lead to the phosphorylation on Ser271 and subsequent activation of 5-LOX by MAPKAP kinases 2 and 3^63^. These findings support a model in which lipolysis triggers neutrophil infiltration through p38-dependent, but chemokine-independent, pathways.

We note that while local IL-1β levels are acutely and transiently increased with sympathetic activation in visceral adipose tissue, levels of the cytokine are also chronically increased in this tissue with obesity^2,7,21,22^. In the latter case, IL-1β is produced by both macrophages and adipocytes due to activation of the inflammasome NLRP3, leading to caspase 1 activation^21,22,64,65^. Interestingly, studies from our laboratory (unpublished) and others^24,66–68^ show that IL-1β and other inflammatory cytokines act to repress energy expenditure in both cases, both through catecholamine resistance^35^ and reduction of AMPK activity^69^. These findings emphasize a common theme in metabolic regulation in which metabolic stress leads to activation of pathways that preserve energy storage in adipocytes and suggest that these pathways might be appropriate targets for metabolic disease.

## Methods

### Ethical statement

The animal study was approved by the Institutional Animal Care and Use Committee (IACUC) at the University of California, San Diego (UCSD). The cell culture study was approved by the Environment, Health and Safety Department at UCSD.

### Animals

Mrp8-cre (strain no.021614) and DTR mice (strain no.007900) were obtained from Jackson Labs. These mice were crossed to generate neutrophil-specific DTR expressing mice (Neu-DTR). Diphtheria Toxin (Sigma, D0564, 20 pg/kg) was injected 24h before CL-316,243 injection. To maintain neutrophil depletion in repeated CL-316,243 injection, DT was injected with CL-316,243. Adipocyte-specific ATGL knockout (adiponectin-driven cre x ATGL fl/fl) mice was a gift from Dr. Elina Zuniga (UC San Diego).

Mice were fed a standard normal chow (7912, Teklad). 8- to 12-week-old mice male mice were used for experiments. Before CL-316,243 treatment, mice were conditioned for intraperitoneal injections for one week. CL-316,243 (0.5 mg/kg) was injected intraperitoneally. VX765 (50 mg/kg, dissolved in 20% cremophor) was injected twice per day. SB 203580 (25 mg/kg, dissolved in 20% cremophor) was injection 30 min before CL-316,243. Mice were housed in a specific-pathogen-free (SPF) facility with 12 h:12 h light:dark cycles and given free access to food and water. All animal use was approved by the Institutional Animal Care and Use Committee (IACUC) at the University of California, San Diego.

### Reagents

CL-316,243 (CL, Sigma C5979), VX765 (MedchemExpress, HY-13205), Diphtheria Toxin (Sigma, D0564), Atglistatin (ATGLi, Sigma, SML1075), Etomoxir (Sigma, 236020), N-Acetyl-L-cysteine (NAC, Sigma, A7250), SB203580 (Selleckchem, S1076), SP600125 (Selleckchem, S1460), mouse recombinant IL-1β (R&D systems, 401-ML), mouse recombinant TNFa (R&D systems, 410-MT), Dexamethasone (Sigma, D4902), DMEM/F-12 50/50 (Corning, 15–090), Fetal bovine serum (FBS, Corning 35–010), Dexamethasone (Sigma D4902), Insulin (Sigma, I6634), 3-Isobutyl-1-methylxanthine (IBMX, Sigma I5879), 1 μM Rosiglitazone (Sigma, 557366).

### Cell culture

3T3-L1 fibroblasts (American Type Culture Collection) were cultured DMEM with 10% NBCS (Newborn Calf Serum). Once grown to 100% confluence, adipocyte differentiation was initiated by adding 500 μM 3-isobutyl-1-methylxanthine, 250 nM dexamethasone and 1 μg/ml insulin in DMEM with 10% FBS for 3 days. The medium was then changed to DMEM with 10% FBS containing 1 μg/ml insulin for 3 days, followed by maintenance in DMEM with 10% FBS. Cells were used for experiments on day 8 or 9 after the initiation of differentiation. Only cultures in which >95% of cells displayed mature adipocyte morphology were used. All media were supplemented with 10 U/ml penicillin, 10 U/ml streptomycin.

Preadipocytes were isolated from inguinal WAT of 6- to 8-week-old mice as previously described^42^. Briefly, dissected fat was finely minced and digested in 5 ml of serum-free DMEM/F12 containing 1 mg/ml collagenase (C6885, Sigma) for 15–25 minutes at 37 °C with gentle agitation. Digestion was stopped by adding FBS to a final concentration of 10%. The tissue suspension was filtered through a 100-μm strainer and centrifuged at 300g for 5 minutes at room temperature. The supernatant was discarded and the pellet was washed once with DMEM/F12 containing 10% FBS, and then resuspended and plated in 10 cm tissue culture plates in DMEM/F12 with 15% FBS. After 2 days, the medium was removed, non-adherent cells were washed away with DPBS. Fresh culture medium containing 10% FBS was added. Once the cells reached ~50 % confluence, they were plated for experiment and maintained in DMEM/F12 with 10% FBS, replaced every 2–3 days. Differentiation was initiated 1–2 days after the cultures reached full confluence by adding 500 μM 3-isobutyl-1-methylxanthine, 250 nM dexamethasone, 1 μg/ml insulin and 1 μM troglitazone for 4 days. The medium was then changed to DMEM/F12 with 10% FBS containing 1 μg/ml insulin for 3 days, followed by maintenance in DMEM/F12 with 10% FBS. Cells were used for experiments on day 8 or 9 after the initiation of differentiation. Only cultures in which >90% of cells displayed mature adipocyte morphology were used. All media were supplemented with 10 U/ml penicillin, 10 U/ml streptomycin.

### Lipolysis

Free fatty acid (FFA) concentrations were measured using 2 μl of serum and the NEFA kit (Wako’s NEFA HR 2, 999–34691- 991–34891- 995–34791- 993–35191), following the manufacturer’s instructions. Blood was collected by cardiac puncture or tail vein, allowed to clot at room temperature for 30 min, and centrifuged at 2,000 g for 10 minutes to isolate serum. Briefly, 150 μl of Reagent A and 75 μl of Reagent B were added, and absorbance was measured at 550 nm with 660 nm as reference. Free glycerol concentrations were measured using either 4 μl of serum or 100 μl of conditioned medium with 100 μl of Free Glycerol Reagent (Sigma, F6428), and absorbance was measured at 540 nm according to the manufacturer’s protocol. All samples are measured by duplicates. Average value was used for analysis.

### ELISA

Serum levels of IL-1β and IL-6 were measured using mouse ELISA kits (R&D Systems, DY401–05, DY406–05) according to the manufacturer’s instructions. Blood was collected by cardiac puncture, allowed to clot at room temperature for 30 min, and centrifuged at 2,000 g for 10 minutes to isolate serum. For each assay, 50 μl of serum was used.

### Ex vivo

At 24 hours after CL-316,243 injection with or without VX765 treatment, mice were perfused with PBS to remove circulating immune cells. Adipose tissues were immediately dissected, weighed, and incubated in KRBH buffer (pH 7.4) at 37 °C in a CO_2_ incubator. Tissues were then cut into 50 mg pieces (approximately 0.5 mm^3^) and placed into individual wells of a 48-well plate containing 0.5 ml DMEM supplemented with 2% BSA. After a 10-minute equilibration period, tissues were treated with either vehicle or CL-316,243. Supernatants were collected after incubation and processed for downstream analysis.

### FACS

To remove circulating neutrophils, mice were anesthetized with isoflurane and perfused with PBS. Adipose tissues were dissected, finely minced, and digested in DMEM containing 1 mg/ml collagenase (C6885, Sigma) at 37 °C for 15–25 minutes with gentle agitation. Digestion was stopped by adding FBS to a final concentration of 10%. The resulting suspension was filtered through a 100-μm cell strainer and centrifuged at 300 g for 5 minutes at room temperature. The supernatant was discarded, and the pellet was resuspended in RBC lysis buffer. After 1 minute of incubation at room temperature, PBS was added to neutralize the lysis. Cells were centrifuged again, and the resulting SVF pellet was resuspended in FACS buffer (1% FBS, 1mM EDTA in PBS). Equal numbers of SVF cells were used across groups for antibody staining. Antibodies: CD16/32 (101302, Biolegend) CD45-FITC (11-0451-82, Biolegend), CD11b-APC.Cy7 (A15390, Biolegend), Ly6G-BV650 (127641, Biolegend), F4/80-PE (12-4801-82, Invitrogen), Ly6C-PE.Cy7 (25-5932-82, Invitrogen), IA/IE-BV785 (107645, Biolegend), Thy1.2-BV421 (140327, Biolegend), CD19-BV421 (115549, Biolegend), Zombie dye (423113, Biolegend). All samples were preincubated with Fc block with Zombie dye for 15 min at 4°C before staining for antibodies for 25 min at 4°C. After staining, samples were fixed in 1% paraformaldehyde and stored in FBS buffer. Flow cytometry data were acquired by ZE5 (Bio-rad) cytometer and analyzed using FlowJo software. Supplementary Fig 1 contains gating strategy. Briefly, single cells were gated at the beginning. There wasn’t overlapping of unique markers of each cell populations (Ly6G, Ly6C, F480) among different cell populations. Neutrophils and monocytes were gated with IA/IE negative at the end to verify their population.

### Histology

Tissues were fixed in 4% paraformaldehyde for 48 hours, then washed and stored in 70% ethanol until processing. Paraffin embedding, sectioning, hematoxylin and eosin (H&E) staining, and immunohistochemistry were performed by the UCSD Tissue Technology Core. Adipocyte size was quantified using Adiposoft in ImageJ and an in-house pipeline developed with CellProfiler^42^.

### Western blotting

Tissues and cells were homogenized in RIPA buffer (50 mM Tris pH 7.5, 150 mM NaCl, 0.1% SDS, 0.5% deoxycholate, and 1% NP-40), supplemented with protease III inhibitor cocktail (Millipore, 539134), phosphatase II inhibitor cocktail (MilliporeSigma, P5726, 5 mL) and phosphatase III inhibitor cocktail (MilliporeSigma, P0044, 5 mL) were added. Homogenates were centrifuged at 17,000g for 15 minutes at 4 °C, and the supernatant was collected. Protein concentration was determined using the BCA assay (Thermo Scientific, 23225). Equal amounts of protein were mixed with Laemmli sample buffer, denatured, and separated by SDS–PAGE. Proteins were transferred onto PVDF membranes (0.22 μm pore size, Bio-Rad) for immunoblotting.

### Antibodies for immunobotting

individual proteins were detected using 1:1000 dilution of primary antibodies, as follows: HSL (4107), pS660 HSL (4126), pS565 HSL (4139), Ly6G (87048), Tubulin (2144), pp38 (4511), p38 (9212), HSP90 (4874), NLRP3 (15101), PKA substrate (9624), β-actin (4967), pJNK (4668), JNK (9252), IκBα (9242). All from Cell signaling. IL-1β (AF-40, R&D systems)

### RNA extraction, RT-PCR

RNA extractions from adipose tissues and cells were performed using TRIzol reagent (Life Technologies, 15596018) followed by purification using the PureLink RNA Mini Kit (Ambion, 12183025). Reverse transcription was carried out using HiScript III 1st Strand cDNA Synthesis Kit (Vazyme, R312–02). Quantitative PCR amplification was performed with Taq Pro Universal SYBR qPCR Master Mix (Vazyme, Q712–02) using the Applied Biosystems QuantStudio 5 Real-Time PCR System. RT-PCR was conducted duplicate or triplicate per same samples. Average value was used for analysis. Sequences of primers used are listed in Supplementary Table.

### RNA seq ana analysis

RNA extraction from primary adipocytes was conducted similarly to the RNA extraction protocol above. One sample from 3 independent experiments was used for each condition (n = 3/group). RNA (100–500 ng) was utilized for library preparation with the TruSeq Stranded mRNA Kit (Illumina) according to the manufacturer’s protocol. Libraries were validated using a 2100 BioAnalyzer (Agilent), then normalized and pooled for sequencing using bar-coded multiplexing at a 90 bp single end read length on an Illumina HiSeq 4000. Samples were sequenced to a median depth of 14 million reads, and fastq files were generated automatically using Illumina bcl2fastq2 Conversion Software. Read alignment and junction mapping to genome build GRCh38 was accomplished using STAR, version 2.7.2b. Known splice junctions from mm10 were supplied to the aligner, and de novo junction discovery was also permitted. Differential gene expression analysis and statistical testing were performed using Cuffdiff2, version 2.2.1, employing the Ensembl genome annotation. Transcript expression was calculated as gene-level relative abundance in fragments per kilobase of exon model per million mapped fragments. Differential expression and gene ontology analyses were conducted using Metascape^70^

### Statistical analysis

All statistical analyses were performed using GraphPad Prism (v10.4.1). Data were shown as mean +/− s.e.m. Comparisons of two groups were assessed using Student’s *t*-test. Comparisons of multiple groups were assessed using One-way or Two-way analysis of variance (ANOVA), followed by Bonferroni’s post-hoc analysis or Turkey’s post-test (indicated in figure legend). P values were indicated on figures. Experimental data was not excluded from the statistical analyses, except for exclusions due to technical errors or loss of confidence in appropriate controls. Measurements were taken from distinct biological samples, except for measuring serum glycerol and NEFA (Fig 4f, g), which was measured longitudinally in the same mice at multiple time points. While processing tissue for RNA extraction, RT-PCR and lysis, investigators were blinded by allocating samples randomly without knowing treatment information. Otherwise, investigators were not blinded during experiments. No estimate of variance was made between each group. There was no predetermination of sample size and sample size was chosen based on available animal or cell numbers. When applicable, mice were randomly assigned to treatment or control groups. Treatment was randomly mixed within same housing cage. All in vivo experiments were conducted with at least two independent cohorts. All in vitro cell experiments were not randomized.

### Schematics

Schematic graphs were created with Biorender.com

## Extended Data

**Extended Fig 7.**
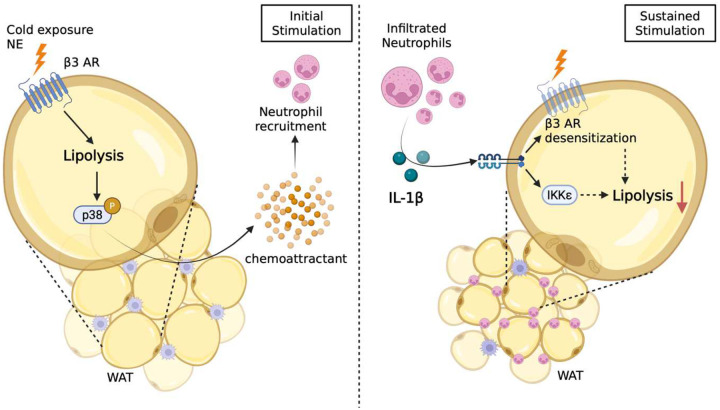
Mechanistic model. β-adrenergic activation transiently recruits neutrophils into white adipose tissue through secreting a lipolysis-p38 dependent chemoattractant. Infiltrated neutrophils secrete IL-1β, which suppresses lipolysis and limits energy loss by inducing catecholamine resistance in adipocytes.

**Extended Table 1.** Primer sequences for analyzing gene expression.

Gene	Forward	Reverse
IL-1β	GCCCATCCTCTGTGACTCAT	AGGCCACAGGTATTTTGTCG
IL-6	AGTTGCCTTCTTGGGACTGA	TCCACGATTTCCCAGAGAAC
Mcp1	TTAAAAACCTGGATCGGAACCAA	GCATTAGCTTCAGATTTACGGGT
Cxcl1	TGCACCCAAACCGAAGTCAT	ACTTGGGGACACCTTTTAGCA
Cxcl2	TCATAGCCACTCTCAAGGGC	TCTTCCGTTGAGGGACAGCA
Tnfa	ACGGCATGGATCTCAAAGAC	AGATAGCAAATCGGCTGACG
Adgre1	CCCCAGTGTCCTTACAGAGTG	GTGCCCAGAGTGGATGTCT
Ucp1	AGGCTTCCAGTACCATTAGGT	CTGAGTGAGGCAAAGCTGATTT
Dio2	AATTATGCCTCGGAGAAGACCG	GGCAGTTGCCTAGTGAAAGGT
Ikbke	ACAAGGCCCGAAACAAGAAAT	ACTGCGAATAGCTTCACGATG
Adrb3	GGCCCTCTCTAGTTCCCAG	TAGCCATCAAACCTGTTGAGC
Ppargc1a	CCACTTCAATCCACCCAGAAA	TATGGAGTGACATAGAGTGTGCT
Cpt1a	ACCAACGGGCTCATCTTCTAA	CAAAATGACCTAGCCTTCTATCGA
CPT2	CAACTCGTATACCCAAACCCAGTC	GTTCCCATCTTGATCGAGGACATC
Acox1	TAACTTCCTCACTCGAAGCCA	AGTTCCATGACCCATCTCTGTC
G0S2	CGCGGATCCATGGAAAGTGTGCAGGAGCTGATCC	CCGCTCGAGTTAAGAGGCGTGCTGCCGGAGGGAC
Arbp	CACTGGTCTAGGACCCGAGAA	AGGGGGAGATGTTCAGCATGT

## Supplementary Material

1

## Data Availability

All data supporting the findings of this study are available within the paper and its Supplementary Information. Source data and uncropped western blot gels are provided with this paper. qPCR primer sequences are provided in Extended Data Table 1.
