# Supplementary material for: Sympathetic activation of white adipose tissue recruits neutrophils to limit energy expenditure
Source: Res Sq. 2025 Apr 16:rs.3.rs-6414640. Preprint. [Version 1] doi: 10.21203/rs.3.rs-6414640/v1 (PMC12047989; doi:10.21203/rs.3.rs-6414640/v1)
Supplement: 1 [file NIHPPRS6414640V1-supplement-1.pdf]

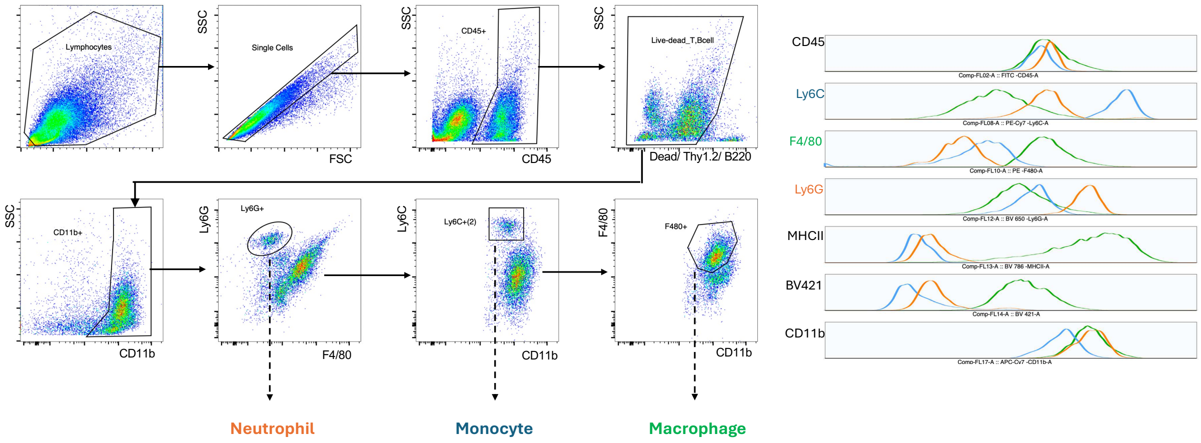

Supplementary Fig 1. Fluorescence-Activated Cell Sorting (FACS) gating strategy. Single cells were gated at the beginning. CD45 positive cells were gated. Among CD45 positive cells, dead, B and T cells were excluded. Then CD11b positive cells were gated. Neutrophils were gated by Ly6G positive cells. Remaining Ly6G negative cells were gated with Ly6C. Ly6C positive cells were defined to be monocytes. Ly6C negative cells were gated for F480 positive cells to defince macrophages. Neutrophils and monocytes were gated with IA/IE. IA/IE negative cells were defined as neutrophils or monocytes.
